# Supplementary material for: Activation of Vasopressin Receptor 1A by Vasopressin Enhances Myometrial Smooth Muscle Cell Excitability by Inhibiting the Potassium Channel SLO2.1
Source: bioRxiv. 2026 Aug 12:2026.08.06.743275. Preprint. [Version 1] doi: 10.64898/2026.08.06.743275 (PMC13640551; doi:10.64898/2026.08.06.743275)
Supplement: Supplementary file 1 [file media-1.pdf]

Supplementary Figures.

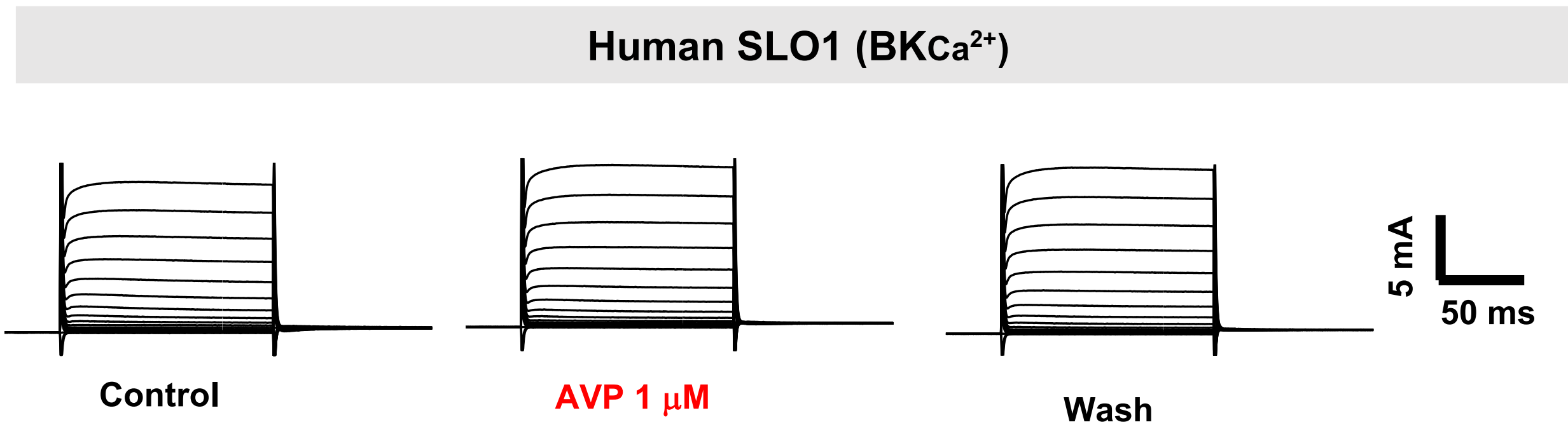

Supplementary Figure 1. AVP does not induce K^+ currents in the absence of AVP receptor. Representative two-electrode voltage-clamp recordings from Xenopus oocytes expressing human SLO1 before and after application of 1 μM AVP.

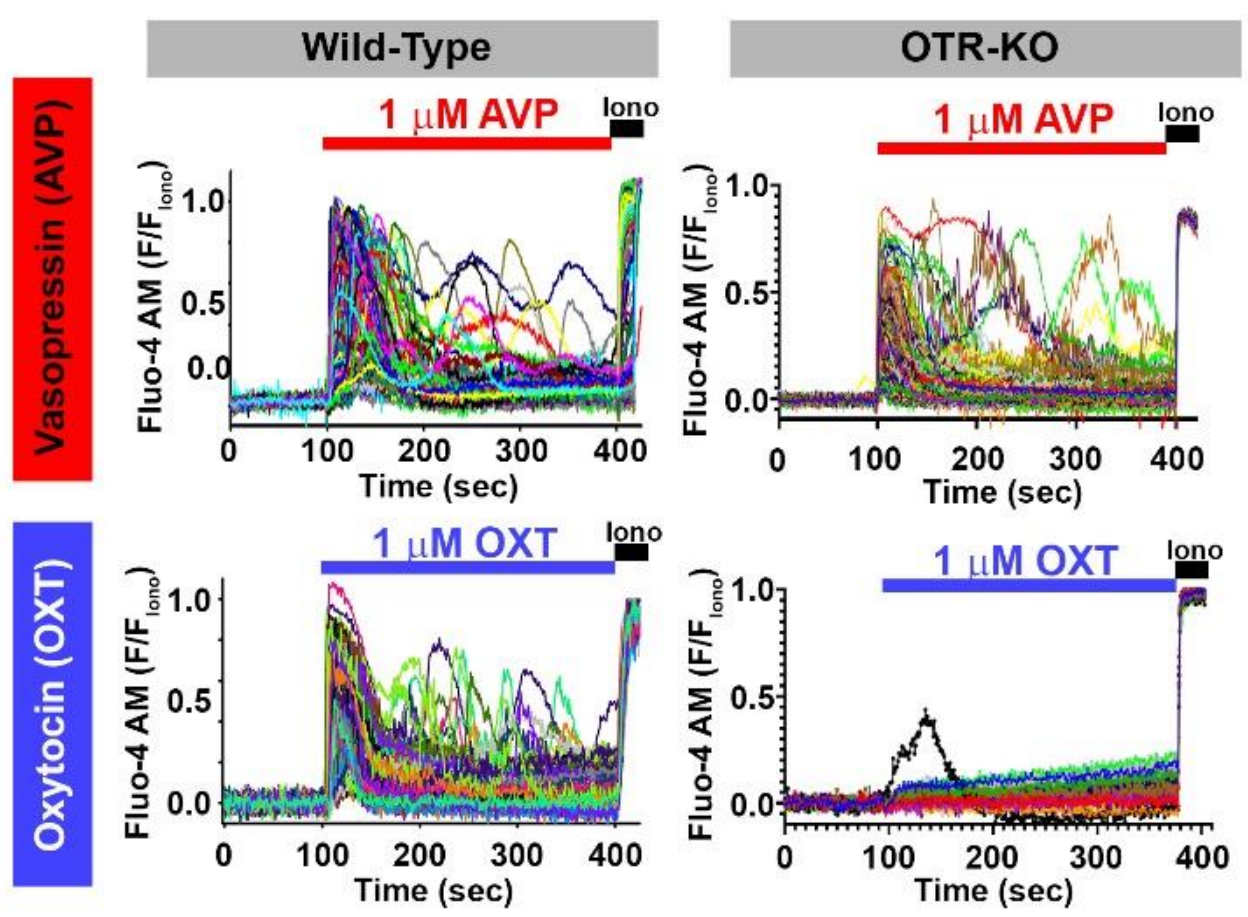

Supplementary Figure 2. AVP and OXT induce Ca^{2+} signals in hTERT-HM Wild type cells and in hTERT OTR KO cells. Representative Fluo-4 AM Ca^{2+} imaging traces from hTERT-HM Wild Type cells **(Top left)** or from Oxytocin receptor KO hTERT-HM Cells (OTR-KO) **(Top right),** both stimulated with 1 μM AVP in the presence of 2 mM extracellular Ca^{2+}**.** Representative Fluo-4 AM Ca^{2+} imaging traces from hTERT-HM Wild Type cells **(Bottom left)** or from OTR-KO hTERT-HM Cells **(Bottom right),** both stimulated with 1 μM OXT in the presence of 2 mM extracellular Ca^{2+}**.** All values were normalized to fluorescence obtained with 5 μM ionomycin (Iono) in 2 mM extracellular Ca^{2+}.
